# Spectral Engineering of Hybrid Biotemplated Photonic/Photocatalytic Nanoarchitectures

**DOI:** 10.3390/nano12244490

**Published:** 2022-12-19

**Authors:** Gábor Piszter, Krisztián Kertész, Dávid Kovács, Dániel Zámbó, Zsófia Baji, Levente Illés, Gergely Nagy, József Sándor Pap, Zsolt Bálint, László Péter Biró

**Affiliations:** 1 Institute of Technical Physics and Materials Science, Centre for Energy Research, 29-33 Konkoly Thege Miklós St., 1121 Budapest, Hungary; 2 Surface Chemistry and Catalysis Department, Institute for Energy Security and Environmental Safety, Centre for Energy Research, 29-33 Konkoly Thege Miklós St., 1121 Budapest, Hungary; 3 Department of Zoology, Hungarian Natural History Museum, 13 Baross St., 1088 Budapest, Hungary

**Keywords:** spectral engineering, biotemplating, hybrid photonic nanoarchitecture, photocatalysis, butterfly wing, ALD, ZnO, Cu_2_O nanoparticles, p-n heterojunction, UV-visible spectroscopy

## Abstract

Solar radiation is a cheap and abundant energy for water remediation, hydrogen generation by water splitting, and CO_2_ reduction. Supported photocatalysts have to be tuned to the pollutants to be eliminated. Spectral engineering may be a handy tool to increase the efficiency or the selectivity of these. Photonic nanoarchitectures of biological origin with hierarchical organization from nanometers to centimeters are candidates for such applications. We used the blue wing surface of laboratory-reared male *Polyommatus icarus* butterflies in combination with atomic layer deposition (ALD) of conformal ZnO coating and octahedral Cu_2_O nanoparticles (NP) to explore the possibilities of engineering the optical and catalytic properties of hybrid photonic nanoarchitectures. The samples were characterized by UV-Vis spectroscopy and optical and scanning electron microscopy. Their photocatalytic performance was benchmarked by comparing the initial decomposition rates of rhodamine B. Cu_2_O NPs alone or on the butterfly wings, covered by a 5 nm thick layer of ZnO, showed poor performance. Butterfly wings, or ZnO coated butterfly wings with 15 nm ALD layer showed a 3 to 3.5 times enhancement as compared to bare glass. The best performance of almost 4.3 times increase was obtained for the wings conformally coated with 15 nm ZnO, deposited with Cu_2_O NPs, followed by conformal coating with an additional 5 nm of ZnO by ALD. This enhanced efficiency is associated with slow light effects on the red edge of the reflectance maximum of the photonic nanoarchitectures and with enhanced carrier separation through the n-type ZnO and the p-type Cu_2_O heterojunction. Properly chosen biologic photonic nanoarchitectures in combination with carefully selected photocatalyst(s) can significantly increase the photodegradation of pollutants in water under visible light illumination.

## 1. Introduction

Emissions of various toxic pollutants from drug residues to dyes from textile industry and communal wastewater to the environment coupled to the emerging global challenges in energy consumption and generation, have led to an exponentially increasing demand for cheap and environmentally harmless ways of water remediation.

Solar energy is a sustainable and de facto inexhaustible energy source for mankind with no environmental concerns. There are several major routes to harness solar energy: photothermal [1], photovoltaic [2,3], and photocatalytic methods [4]. Nanostructures and nanocomposites are frequently used to enhance solar energy conversion [5,6,7,8]. For example, plasmonics allow extraordinary control of light, making it attractive for application in solar energy harvesting. In metal−semiconductor heterojunctions, plasmons can enhance photoconversion in the semiconductor via three mechanisms, including light trapping, hot electron/hole transfer, and plasmon-induced resonance energy transfer (PIRET) [5]. Photonic crystals can enhance the interaction of light with a semiconductor. Integrated photonic crystals and the plasmonic effects of nanostructured materials may have an enhancing superposition effect in controlling light [6].

Photocatalysts and photovoltaics represent two major approaches sharing similar processes (including light absorption, charge carrier generation, and separation) for solar energy conversion with semiconductors. Integrating semiconductors with plasmonic nanostructures was proven as an effective way to greatly enhance the performance in photocatalysis and photovoltaic devices [8]. Adding photonic crystals—capable of controlling light propagation—can further enhance this process. Inverse opal type photonic crystal (PhC) nanoarchitectures were used to host various catalytic nanoparticles (NPs) [9]. Slow light effects [10] and vibrational strong coupling of molecules inside a cavity [11,12] have much potential for molecular and materials sciences that is just beginning to be explored. The hybrid nanoarchitectures (PhC + NP) exhibit a vast potential for harnessing solar radiation for catalytic purposes and offer a further advantage as the PhC component may provide a supporting substrate with a large specific surface for the catalytic NPs [13]. The solar spectrum reaching the Earth’s surface is attenuated by atmospheric absorption; the peak power output is in the visible range; therefore, materials suitable for this spectral range are the most useful for potential applications. UV-transparent materials may have prohibitive prices for wastewater treatment, for example.

The cheap fabrication of large area and good quality three-dimensional (3D) PhCs is still very challenging [14]. On the other hand, biological evolution offers a rich “library” of PhC-type nanoarchitectures on the wings of butterflies exhibiting structural coloration [15,16,17,18,19,20,21,22]. These are nanocomposites of chitin and air producing color in the near UV and visible spectral range. Usually, they have air-filled nanocavities that can host metallic or semiconducting NPs. Here, it is worth emphasizing that moths, such as the domesticated mulberry moth (*Bombyx mori*), have been used for thousands of years [23] to produce natural silk. The production of natural silk amounts to approximately 80,000 tons/year [24]. It was shown recently that it is possible to obtain more than 600 exemplars of *Polyommatus icarus* from a single breeding pair [13,25] in laboratory conditions. In the experiments reported in the present paper, we used wings of male *P. icarus* butterflies resulting from this type of breeding. Therefore, the wings of butterflies exhibiting structural color may constitute a cheap and ready-made “library” to experiment with biotemplating and the effects of spectral engineering on the biotemplated photonic nanoarchitectures.

We were interested in exploring the possibilities offered by using standard materials science methods for the spectral engineering of the photonic/photocatalytic nanoarchitectures, such as NP sol preparation and drop-drying on the biologic photonic nanoarchitectures, atomic layer deposition (ALD), and their combinations, to tune the structural color of butterfly wings in a controlled way and to explore the effects of this tuning on the photocatalytic properties of the biotemplated hybrid nanoarchitectures. As test materials, we chose octahedral Cu_2_O nanoparticles and ZnO coating by ALD. Cu_2_O NPs are intensely investigated p-type semiconductor NPs with characteristic absorption in the visible light spectrum [26], while ZnO is also a well-studied n-type photocatalytic semiconductor [27], but unfortunately, its absorption edge is at the limit of the UV to visible light range [28]. Various Cu_2_O and ZnO heterojunctions were also investigated [29,30,31], including self-powered sensing devices [30,32]. The Cu_2_O-ZnO heterojunction extends the absorption of the nanocomposite to the visible range and provides efficient charge separation [33,34,35,36]. Both materials are cheap, abundant, and nontoxic.

Recently, it was reported that the photocatalytic enhancement obtained with TiO_2_ coated nanoporous anodic alumina distributed Bragg reflectors is at the maximum when the red edge of the photonic stop band is spectrally close to the red or blue boundary of the absorption band of methylene blue (MB) and dramatically decreases within the absorbance maximum of MB due to light screening by dye molecules [37]. This, together with our recent finding that the overlap between the reflectance peak of the photonic nanoarchitecture and the dye absorption determines the efficiency of rhodamine B (RhB) decomposition on biotemplated ZnO photonic nanoarchitectures [38], clearly shows the importance of spectral engineering [39,40,41] in photocatalytic applications.

In the present work, we explored the possibilities of enhancing the decomposition of the RhB dye by the spectral engineering of the optical properties of composite photocatalytic surfaces. For this purpose, hierarchical, hybrid biotemplated photonic nanoarchitectures were constructed from butterfly wings—ZnO conformal coating—Cu_2_O nanoparticles. To exploit the slow light effect, we aimed at achieving a good overlap between the red edge of the reflectance peak of the photonic nanoarchitecture and the absorption peak of the dye. The methods of spectral engineering using various combinations of the different elements and the resulting photocatalytic performances of the samples are discussed.

## 2. Materials and Methods

The wings of the Palearctic Blue butterfly, *Polyommatus icarus* (Rottemburg, 1775) (Arthropoda: Insecta: Lepidoptera: Lycaenidae) were used. This butterfly species is not subjected to any restrictions. The range of the species covers the entire Palearctic region [42,43], and recently, their presence was reported in the Nearctic, too [44,45]. Limited color differences exist between Europe and Asia (of the order of 20 nm in the spectral position of the blue reflectance maximum) [46], and the color variations in the blue dorsal color of the males within a given population are of the order of only ±10 nm in the spectral position of the reflectance maximum [47]. The color is stable on time scales of the order of hundreds of years [46]. Wings of butterflies reared in an insectarium with controlled light and environmental conditions [13,25] were used. After drying their wings, the butterflies were frozen and dried for several months separately in small plastic boxes.

**Figure 1:**
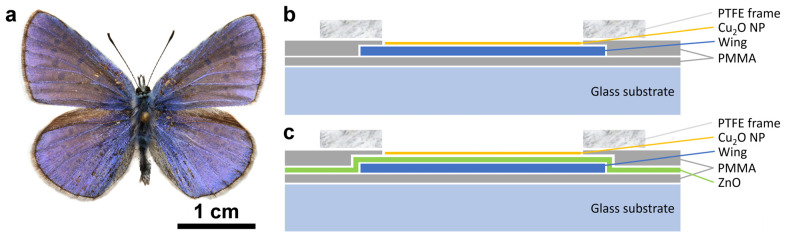
Male *Polyommatus icarus* butterfly and typical sample structures used. (**a**) Dorsal wing surfaces of a male *P. icarus* specimen. Note the homogeneous blue coloration over the entire surface of the four wings; (**b**) Type-1 sample structure without 15 nm ZnO layer on the butterfly wing before the Cu_2_O deposition; (**c**) Type-2 sample structure with 15 nm ZnO layer deposited by ALD on the butterfly wing before the Cu_2_O deposition. Note that the various layer thicknesses are not drawn to scale.

The dorsal wing surfaces of male *P. icarus* butterflies (Figure 1a) were used as biological photonic nanoarchitectures colored by structural color. Two different groups of sample structures were used: type-1 samples without (Figure 1b), and type-2 samples with (Figure 1c) 15 nm of conformally deposited ZnO layer [38].

For octahedral Cu_2_O nanoparticles, slight modifications and upscaling were applied on a recently published protocol [48]. Briefly, 1 mL Cu(NO_3_)_2_ solution (0.1 M) was added to 91.28 mL ultrapure water in a Schott glass and stirred for several minutes. Upon introducing the base solution (200 μL of 1 M NaOH), the solution turned light blue due to the formation of Cu(OH)_2_, which was reduced by the swift addition of hydrazine solution (3 mL, 0.2 M) under vigorous stirring. The solution turned orange within the first minute, and the growth proceeded for 10 min. The particles were collected and washed via centrifugation and redispersion with ethanol–water mixtures (50:50 *V*/*V*%), and finally, the Cu_2_O nanoparticles were redispersed in 105 mL of absolute ethanol to prepare the solution for deposition experiments.

Type-1 samples were prepared according to the following sequence: all four wings were detached from the body of the butterfly. The wings were glued onto glass slides using a very thin layer of polymethyl methacrylate (PMMA). After the gluing, the samples were left to dry overnight; reflectance spectra were measured the next day. An integrating sphere was used for the reflectance measurements to reduce the effects arising from wing scale orientation with respect to the wing plane (see Figure 10 in [47]). After the measurement, three of the glass-mounted samples were left overnight in ethanol and gently washed with fresh ethanol before drying. A third sample was kept for comparison. To avoid the uncontrolled spreading of the Cu_2_O sol, 1 mm thick polytetrafluoroethylene (PTFE) frames with a circular opening of 8 mm in diameter (Figure 1b) were glued carefully—also using PMMA—onto the glass mounted wings in such a way to avoid the flowing out of the PMMA solution into the circular opening. The samples were left to dry overnight, and reflectance measurements were carried out the next day, placing the sample port of the integrating sphere onto the PTFE frame. After the reflectance measurement, the Cu_2_O sol in ethanol was carefully added into the circular opening of the PTFE frames in doses of 40 µL. Samples with 40, 80, and 120 µL were prepared. After adding the amount of 40 µL, the next dose was added only after the complete drying of the sample.

Type-2 samples were prepared according to the following sequence: all four wings were detached from the body of the butterfly. For one butterfly, two wings were kept in pristine state as comparison samples, the other two wings were used to prepare samples for further processing, these wings were fixed onto a Si wafer and loaded in the ALD apparatus. Next, a 15 nm conformal ZnO layer was deposited onto the wings. The homogeneity of the deposition was checked using a photo-processing procedure reported earlier [49] based on the transformation from the RGB color space to HSB through the parameters of hue, saturation, and brightness, which provides a more straightforward and objective way to analyze such photographs. The processed wing images are shown in Figure S1. After the ALD, the samples were glued with PMMA onto glass substrate and left to dry overnight; reflectance spectra with the integrating sphere were measured the next day. After measuring the spectra, the same procedure was used for the type-2 as for the type-1 samples, starting from the gluing of the 1 mm thick PTFE frames.

Similar samples were prepared only using bare glass slides with the PTFE frames with the 8 mm circular opening. These samples were used to test the spreading of the sol and the uniform distribution or clustering of the Cu_2_O.

Another type of samples was prepared on filter paper. A small piece of filter paper was fixed horizontally to the tip of a tweezer and the Cu_2_O sol was added in amounts of 40 µL.

The atomic layer deposition of the ZnO films took place in a Picosun Sunale R-100 (Espoo, Finland) ALD reactor at 100 °C with an 8 hPa background nitrogen pressure. The precursors were diethylzinc (DEZ) from Strem Chemicals (Bischheim, France) and 18 MΩ high purity DI water. The pulse lengths were chosen long enough so that the precursors may have time to diffuse into and homogeneously cover the pores of the structure (0.5 s pulses, with 15 s purging after the DEZ pulse and 20 s after the water pulse).

Optical reflectance and transmittance measurements were carried out by means of an Avantes (Apeldoorn, The Netherlands) modular fiber-optic system. We used an AvaSpec-HERO spectrometer, an AvaLight-DH-S-BAL stabilized UV–visible light source, an AvaSphere-30-REFL integrating sphere, and for reference, a WS-2 white diffuse tile. When necessary, a 5-point smoothing with FFT filter was applied, and the datasets were plotted with OriginPro 2021 (OriginLab Corporation, Northampton, MA, USA).

The presented optical micrographs and the images with extended depth of focus (EDF) were obtained using a Zeiss Axio Imager A1 (Jena, Germany) and a Nikon Eclipse LV150N (Nikon Instruments, Tokyo, Japan), respectively.

For scanning electron microscope inspection, a few mm^2^ wing pieces were cut and fixed on metallic sample holder stubs with conductive tape. In order to maintain the original state of the samples, no other treatment was applied. Images were taken using Thermo Fisher Scientific Scios 2 DualBeam (Waltham, MA, USA) device.

To test the effects of spectral engineering on the photocatalytic activity of the samples, rhodamine B (RhB) was used. The photocatalytic decomposition experiments were carried out in a glass cuvette 5 × 5 × 1 cm^3^ containing 20 cm^3^ of 15 μM RhB dissolved in water. A heat-free Asahi Spectra MAX-301 (Torrance, CA, USA) Xe lamp (300 W) with fiber optics was applied as a light source supplying a square-shaped beam (1.5 cm × 1.5 cm). The lamp-to-catalytic surface distance was 6 cm (corresponding to ca. 100 mW cm^−2^ light power). The conversion of RhB was followed by detecting the change in the characteristic absorption band at 554 nm vs. time using an Agilent Cary 60 (Santa Clara, CA, USA) UV–Vis spectrophotometer equipped with an immersion probe (*l* = 1 cm) that was placed inside the solution out of the illumination area. The conversion was calculated as (A_0_ − A_i_)/A_0_, where A_0_ is the initial absorbance of RhB at 554 nm, and A_i_ is the absorbance at a given point in the reaction. The solution was continuously stirred during the reaction. The cuvette wall reached 80% transmittance at the wavelength of 360 nm. The active photocatalytic surface was a circle of 8 mm in diameter. The experimental conditions were chosen in a way that the ratio of the volume of RhB solution to the photocatalytic surface provided quasi-stationary reaction conditions throughout each experimental run for a better comparison of the initial rates. Soaking tests in RhB solution ruled out any detectable role of initial adsorption of the dye in the change in absorbance (note the high reaction volume over catalytic surface area ratio).

## 3. Results

**Figure 2:**
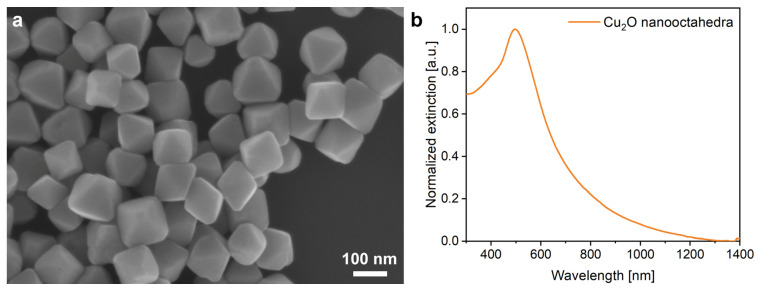
Cu_2_O nanoparticles stored in ethanol. (**a**) SEM micrograph of the nanoparticles drop dried on Si; (**b**) Extinction of the nanoparticles suspended in ethanol.

The Cu_2_O nanoparticles were of octahedral shape and uniform size (Figure 2), with the base edge length in the range of 136 (±12) nm and the maximum of their extinction is at 505 nm, which corresponds to their collective scattering and the band-edge absorption. Due to the smooth facets of the nano-octahedra, the extinction spectrum consists of a single narrow peak.

**Figure 3:**
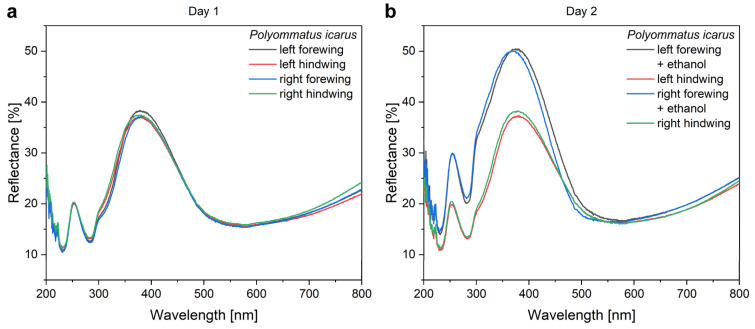
The effect of ethanol pretreatment and the reproducibility of the spectral measurements exemplified by the four wings of the same butterfly. (**a**) The reflectances of the four glass mounted wings measured before the ethanol pretreatment are shown, while in (**b**), the spectra of the same four wings, remeasured next day, are shown after two of the wings have undergone the ethanol pretreatment.

The ethanol pretreatment of the wings was applied to avoid the uncertainty that a certain modification in the optical properties is caused by the ethanol or the nanoparticles. The effect of the ethanol pretreatment is shown in Figure 3.

**Figure 4:**
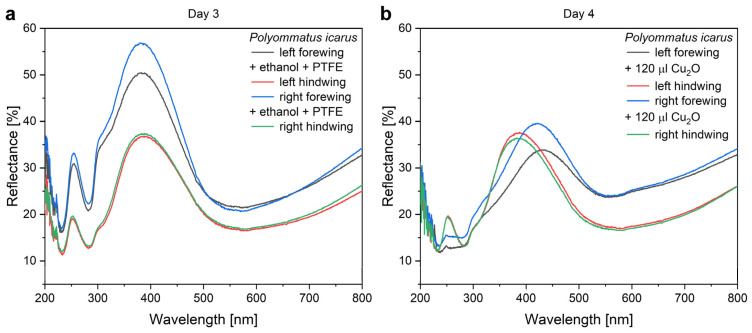
The modification of the optical properties of type-1 samples after the application of 120 µL of Cu_2_O sol onto the wing. (**a**) Two wings after PMMA gluing onto glass, two wings after the ethanol pretreatment followed by mounting of the PTFE frame. (**b**) The ethanol-pretreated wings received the 3 × 40 µL Cu_2_O sol.

**Figure 5:**
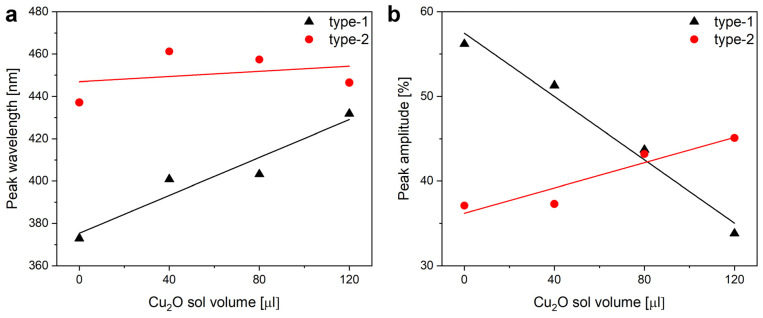
Modifications of the optical properties of the type-1 and of the type-2 samples after the application of the increasing amounts of the Cu_2_O sol onto the wing. (**a**) Peak wavelength and (**b**) peak intensity values are shown with their linear fits to guide the eye.

The application of increasing amounts of Cu_2_O sol on the wings when preparing type-1 samples produced monotonic changes in the optical properties of the wings. The nanoparticles always redshifted the reflectance maximum of the wings. The magnitude of the redshift increased with the increasing amount of nanoparticles on the wing. The redshift was associated with the decrease in the intensity of the reflectance maximum (Figure 4). The modifications of the optical properties of the type-1 and of the type-2 samples upon the application of the Cu_2_O sol are summarized in Figure 5.

**Figure 6:**
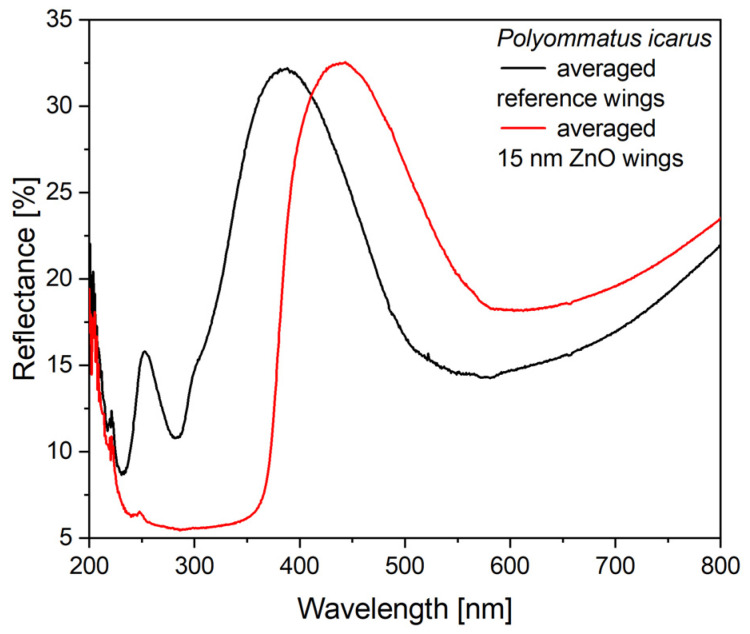
The effect of the deposited 15 nm thick ZnO layer onto the wings of *P. icarus* males.

In the case of the type-2 samples, the Cu_2_O sol was applied on wings conformally covered by 15 nm ZnO layer deposited by ALD. The ZnO deposition modified the optical properties of the wing as shown in Figure 6. The most relevant modifications are: (i) the spectral position of the reflectance maximum is redshifted by 50 nm to 438 nm; (ii) due to the absorption of the ZnO layer, the samples have a very low reflectance below 380 nm.

**Figure 7:**
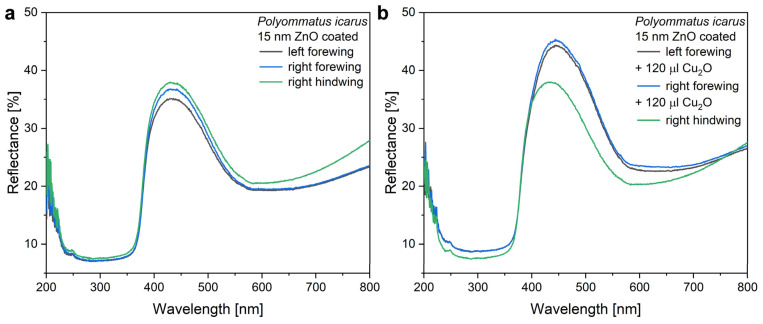
Change in the reflectance of two *P. icarus* males’ wings conformally covered by 15 nm ZnO after the application of 120 µL of Cu_2_O sol. (**a**) Reflectance of the 15 nm ZnO coated wings and (**b**) reflectance of the same wings after the application of 120 µL Cu_2_O sol.

The modifying effect of 120 µL of Cu_2_O on the reflectance of a wing with the 15 nm ZnO coating is shown in Figure 7, while in Figure 5, the modifications induced by the application of 40, 80, and 120 µL of Cu_2_O sol on type-1 and type-2 samples are compared. One may observe that the application of the increasing amounts of Cu_2_O sol onto the type-2 samples has a smaller effect on the optical properties of these samples than was found for type-1 samples.

**Figure 8:**
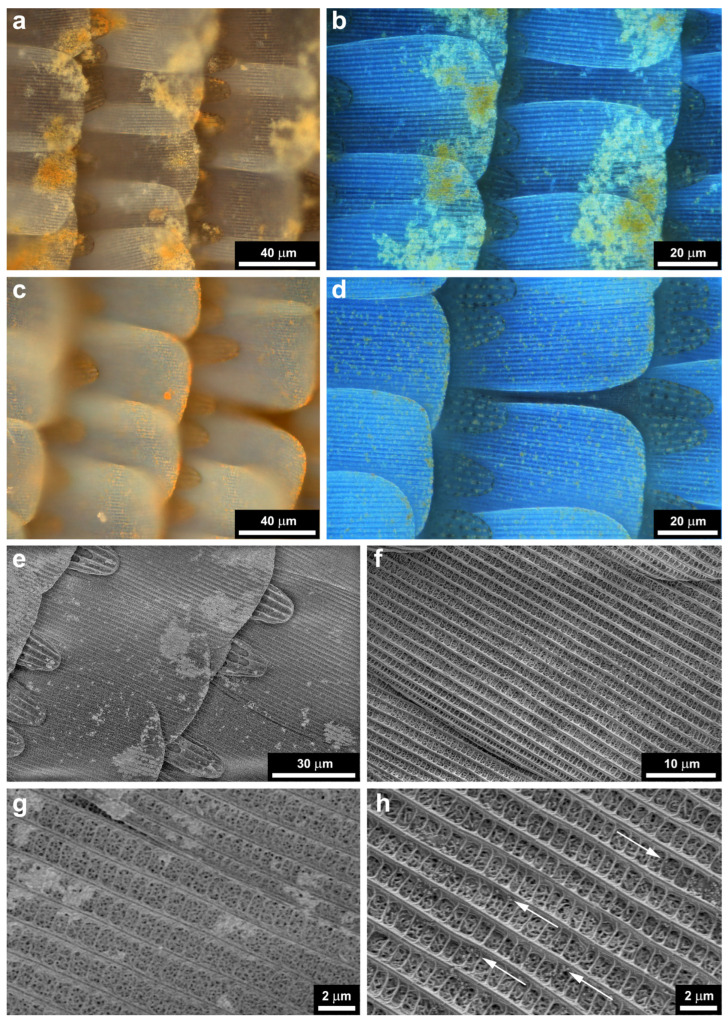
Optical and SEM micrographs of type-1 and type-2 samples after the application of 120 µL of Cu_2_O sol. (**a**) Type-1 sample, optical microscope image recorded with crossed polarizers; (**b**) type-1 sample image recorded on a focus stacking optical microscope using partially crossed polarizers; (**c**) type-2 sample, optical microscope image recorded with crossed polarizers; (**d**) type-2 sample image recorded on a focus stacking optical microscope using partially crossed polarizers; (**e**) SEM micrograph of a type-1 sample; (**f**) SEM micrograph of a sample type-2; (**g**) detail of image in (**e**), note the clustering between the ridges; (**h**) detail of the image in (**f**), note the individual nanoparticles between the ridges marked by arrows.

The optical microscopy images and the SEM micrographs of type-1 and type-2 samples are shown in Figure 8. The major difference is that on the type-1 samples, the Cu_2_O nanoparticles presented a much stronger tendency of clustering compared with the type-2 samples, where a much more uniform distribution of individual nanoparticles is found.

**Figure 9:**
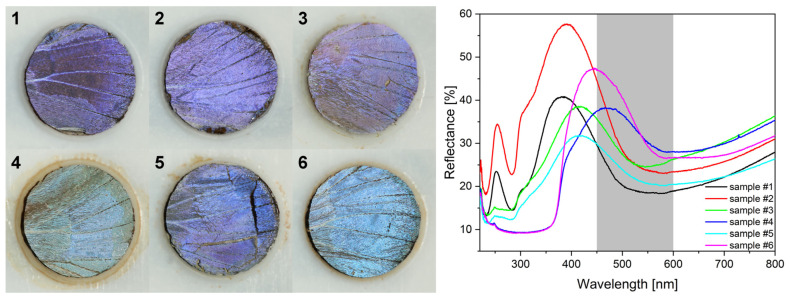
Photographs (**left**) of the wing samples used in the photocatalytic experiments in as-prepared state and the corresponding reflectance spectra (**right**): (**1**) type-1 sample without ethanol pretreatment; (**2**) type-1 sample with ethanol pretreatment; (**3**) type-1 sample with 120 µL of Cu_2_O sol drop dried; (**4**) type-2 sample with 120 µL of Cu_2_O sol drop dried; (**5**) type-1 sample with 120 µL of Cu_2_O sol drop dried, followed by the deposition of 5 nm of ZnO; (**6**) type-2 sample with 120 µL of Cu_2_O sol drop dried, followed by the deposition of 5 nm of ZnO. The grey-shaded area marks the rhodamine B absorption band.

**Figure 10:**
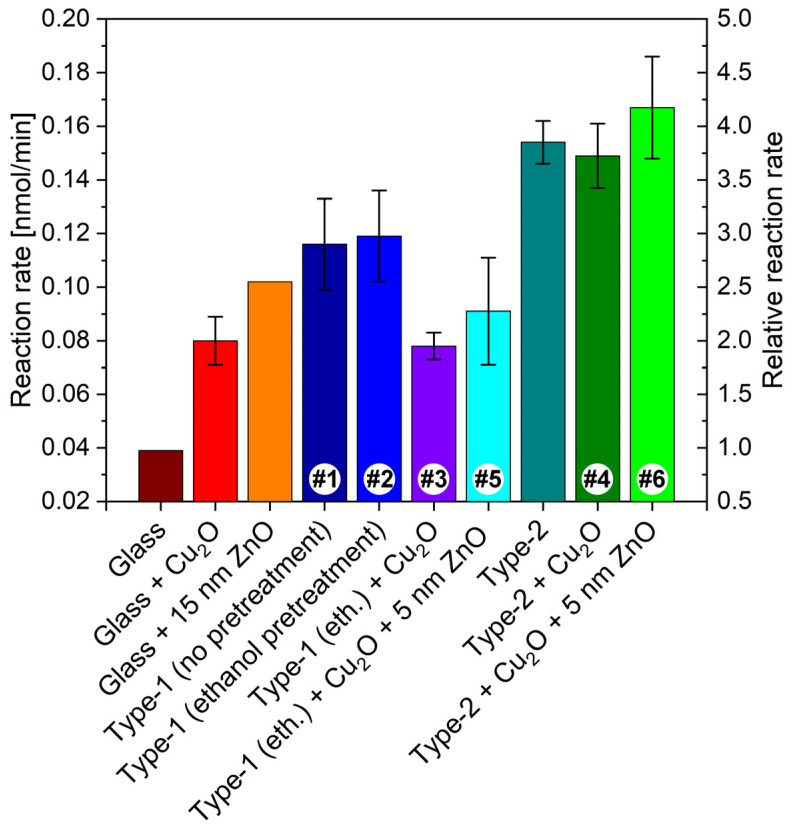
Absolute and relative reaction rates of the samples used to characterize the photocatalytic efficiency of the biotemplated photonic/photocatalytic nanoarchitectures. The value corresponding to bare glass was taken as unity for the calculation of the relative rates. The sample numbers on the bars are the same as those in Figure 9.

In Figure 9, the digital photographs of the wing region seen through the circular opening of the PTFE frame are shown for type-1 and type-2 samples before the first photocatalysis experiment. Color differences are in agreement with the reflectance spectra shown in Figure 9. The color of the samples and the corresponding spectra after the first photocatalytic experiments followed by gentle washing to remove RhB are presented in Figure S4. All samples exhibit modified color after the photocatalytic testing. The modifications are associated with the presence of adsorbed RhB. The averaged reaction rates of RhB photodegradation are summarized in Table 1, and the relative reaction rates are compared in Figure 10.

The distribution of Cu_2_O nanoparticles on glass slides and glass slides covered by 15 nm ZnO masked with similar PTFE frames to avoid the uncontrolled spreading of the sol was investigated by optical microscopy and by spectral measurements, as in Figure S2. The effect of particle concentration on the light scattering was also investigated by combining integrating sphere and standard transmittance measurements, seen in Figure S2. From these measurements, one may observe that the Cu_2_O NPs both on glass and ZnO-covered glass exhibit absorption extended into the visible range to 600 nm with the maximum in the range of 450 nm and are responsible for increasing light scattering as the number of NPs increases.

To examine if the optical properties of the Cu_2_O nanoparticles in a dry state in air correspond with the extinction measured in ethanol (Figure 2), a nanoparticle loaded filter paper was measured (Figure S3). Filter paper was chosen because its major component, cellulose, has a chemical formula close to chitin, the major component of butterfly wings. Furthermore, in the filter paper, the NPs are arranged in a 3D fiber network not on a flat surface. A piece of filter paper was placed in the Cu_2_O sol and stirred magnetically. By dividing the reflectance of the Cu_2_O loaded filter paper by the reflectance of a pristine piece of filter paper, the reflectance minimum was obtained at 450 nm, slightly blue-shifted due to the different medium surrounding the NPs, as compared with the extinction maximum at 500 nm obtained for the NPs suspended in ethanol. In Figure S3, the gradual decrease in the reflectance in the range of 200 to 600 nm with the increase in the amount of the Cu_2_O NPs loaded on the filter paper is shown. In this experiment, the same piece of filter paper with a size comparable with the window in the PTFE frame was kept horizontally and increasing amounts of sol were loaded onto it. After each loading–drying cycle, spectra were taken. In agreement with the data of Figure S2, absorption extended into the visible light spectrum to 600 nm was found, with the maximum in the range of 450 nm.

## 4. Discussion

Heterogenous photocatalysis is a light driven process that enables transformation of the abundant and environmentally safe sunlight into much needed chemical processes to achieve, for example, water purification. For this purpose, supported catalysts are needed, which can be used in a continuous-flow regime. To enhance the efficiency of the purification process, the properties of the used catalysts have to be tunable in a way to fit the characteristics of the pollutant to be removed. For this type of application, the photocatalyst has to be cheap and, in order to allow avoidance of the use of UV transparent materials with prohibitive prices, preferably, able to operate with visible light [50,51].

Due to their nanostructure, butterfly wings offer a support with a large specific surface that can be produced in a cheap and environmentally safe way, onto which different NPs can be immobilized and eventually coated by a few nanometers of different materials to enhance their catalytic effect. An additional benefit may arise from the photonic-crystal-type structures in the butterfly wings. To reveal these possibilities, we used the *P. icarus* wing—Cu_2_O NPs—ZnO coating system. In Figure 3 and Figure 4, the “spectral history” of the same four butterfly wings is presented during a period of four days of processing for type-1 samples. All the samples were re-measured each day. This clearly shows the reproducibility of the measurements and the modifications induced by each processing step. Initially, all four wings possessed overlapping reflectances. The ethanol pretreatment increased the reflectance and induced a slight blueshift of the reflectance maximum together with the broadening towards the UV. This is attributed to the dissolution of a thin layer of waxy material that coats the chitinous scales conformally [52,53,54]. The wax is present to make the wings superhydrophobic [55]. The pretreatment in ethanol removes a few nanometers of the wax layer, therefore, inducing a modification similar to that produced by oxygen plasma treatment [56].

The mounting of the PTFE frames (Figure 4) induces some differences in the intensity of the reflectance of the pretreated wings but without any spectral shift. The application of 120 µL of Cu_2_O sol onto the wings shifts the position of the reflectance maximum towards the red and reduces the intensity of the reflectance maximum. The reduction in the intensity is of a similar magnitude to that found on the filter paper in Figure S3 and is attributed, on one hand, to the absorption of the Cu_2_O and to the addition of a high refractive index component to the photonic nanoarchitectures, as it was found for Al_2_O_3_ deposition on *P. icarus* wings [56]. The shift towards the red side of the visible range is opposite to the modification induced by the ethanol pretreatment, indicating that the NPs do not act as a “filter” but constitute, together with chitinous photonic nanoarchitecture, a new, hybrid photonic nanoarchitecture with modified optical properties [13]. Opposite to the ethanol pretreatment, the application of the Cu_2_O NPs onto the butterfly wing broadens the reflectance maximum towards the red. Due to the increased absorption, there is a tradeoff between the spectral shift produced and the decrease in the reflectance intensity (Figure 5). A good balance between this tradeoff and the desired spectral position can be easily found by choosing a butterfly species with color close to the desired spectral range.

When preparing type-2 samples, the first step was the conformal coating of the wing with 15 nm of ZnO by ALD. The effect of this coating on the reflectance spectrum is shown in Figure 6 and the good uniformity in the color of the coated wings can be seen in Figure S1. It is worth pointing out that the ZnO coating does not reduce the intensity of the reflectance maximum, only shifts its spectral position towards the red. Opposite to Cu_2_O, the absorption edge of ZnO is close to 380 nm and it does not absorb energy from the visible light spectrum. Due to this absorption edge, the reflectance of the ZnO-deposited wings shows a sharp drop in the blue (Figure 6). When the 120 µL of Cu_2_O sol is drop-dried onto these wings, some redshift of the reflectance maximum is observed, but the magnitude of this redshift is reduced compared with the application of the same amount of sol on the uncoated wings (Figure 7). The sharp cutoff in the blue is preserved. The reduced redshift for the type-2 samples is attributed to thickening already produced in the high refractive index component of the photonic nanoarchitecture by the 15 nm of conformal ZnO deposition [56]. The second reason for the smaller effect is the non-uniform thickening on the initial ZnO layer by the addition of Cu_2_O on type-2 samples. The Cu_2_O nanoparticles locally increase the thickness of the high refractive index component. This way, the fraction of the high refractive index component will be shifted even further from the initial biological optimum determined by millennia of evolution. In extremis, this process can lead to the complete annihilation of the photonic crystal type reflectance.

In Figure 7b, the samples treated with Cu_2_O exhibit some increase in reflectance compared with the untreated sample. When looking at the data in Figure 5 comparing the spectral and intensity modifications induced on samples of type-1 and type-2, one can observe that the conformal covering of the wings by the 15 nm thick ZnO layer reduces the modifications produced by the various amounts of Cu_2_O sol to a great extent. For the type-2 sample, only a weak trend can be observed in the position of the reflectance maximum, while for the type-1 samples, the Cu_2_O causes monotonic increase (spectral position) or monotonic decrease (intensity) of the spectral characteristics. The cause of this weak trend for the type-2 samples is the already induced modification by the ZnO conformal coating, which, for example, already shifted the spectral position of the reflectance maximum in the position where it will be shifted for a type-1 sample after the application of 120 µL of Cu_2_O sol. The slight increase in the intensity of the reflectance in Figure 5b for those type-2 samples, which received increasing amounts of the Cu_2_O NPs, may be due to the increased light scattering (compare Figure S2d and Figure S2f) by the Cu_2_O NPs uniformly distributed over the wing (Figure 8). The integrating sphere picks up all the backscattered light towards the side from which the sample is illuminated.

After the application of 120 µL of Cu_2_O, a group of type-1 and type-2 samples were subjected to the deposition of an additional layer of 5 nm of ZnO by ALD in the same deposition run. The purpose of this treatment was threefold: (i) to improve the adherence of the Cu_2_O nanoparticles so that they can withstand the stirring of the test reaction medium, or flow—having in mind real life applications; (ii) to improve the electrical contact between the Cu_2_O nanoparticles and the 15 nm ZnO layer if this was present (type-2); (iii) to test if this thin ZnO layer is sufficient to enhance the charge transfer at the Cu_2_O–ZnO junction, when no previous deposition of 15 nm ZnO onto the wings was performed (type-1). The effects of the deposition of 5 nm of ZnO on the reflectance can be seen in Figure 9. This additional ZnO deposition had the opposite effect on type-1 and type-2 samples: the intensity of the reflectance decreased for type-1 and increased for type-2 samples, and a small blueshift was also present.

The careful investigation of type-1 and type-2 samples, which received 120 µL of Cu_2_O NP sol deposition, revealed clear differences in the spreading of the NPs on the wing scales. In Figure 8a,c, the type-1 and type-2 samples are shown under the optical microscope with crossed polarizers to enhance the visibility of the NPs. It can be clearly seen that on type-1 samples, a pronounced tendency for clustering of the NPs is observed towards the rounded end of the scales, while on type-2 samples, a more uniform distribution can be seen. These observations are supported by the images taken with a focus stacking microscope to compensate for the tilt of the scales with respect to wing membrane (the sample plane) (Figure 8b,d). The differences in the distribution of the NPs on the type-1 and type-2 samples are attributed to the different surface chemistry of the two kinds of samples. The SEM images of Figure 8 fully support the optical microscopy observations. In the detailed images in Figure 8g,h, one can see that while on the type-1 sample, the smaller grouping of the NPs also shows clustering, on the type-2 sample, individual NPs can be seen.

In Figure 9, the digital photographs of the samples used for the catalytic experiments and their corresponding spectra are shown. Even by the naked eye one can perceive the color differences in the samples. In decreasing order, samples #6, #2, and #4 have the highest value of reflectance at the blue edge of the RhB absorption. As was reported recently [37,57,58], the blue and the red edge of the dye absorption are the most efficient for photocatalysis enhancement by the slow light effect, because in the central part of the absorption band, the screening by the dye itself greatly reduces the intensity of the light falling on the photonic nanoarchitecture. If comparing the catalytic performance of these samples (Figure 10) with the ordering of the reflectance intensities, one can observe that despite the nearly identical intensity of sample #6 and #2 at the blue edge of the RhB absorption edge, their catalytic performance is different. This clearly shows that not only the optical properties but the type of the photocatalytic coating is also important for the faster reaction rate.

Three other samples worth comparing in Figure 9 are #1, #3, and #5. Samples #1 and #5 have the same reflectance intensity at the blue edge of the dye absorption, but both samples #3 and #5 are less efficient in the photodecomposition of the RhB than the pristine butterfly wing. In other words, the application of Cu_2_O NPs on the butterfly wing deteriorated the photocatalytic efficiency (Figure 10). Some improvement is produced in sample #5 by coating the Cu_2_O NPs on the wing by 5 nm of conformal ZnO (Figure 10). A slight reduction in the reaction rate is observed on the type-2 sample, also, after the application of the Cu_2_O NPs, but the reaction rate improves after the additional 5 nm ZnO was deposited on the sample (Figure 10). These observations underscore the importance of the type of the semiconductor, which is in contact with the dye, as Cu_2_O is of p-type and ZnO is of n-type. In the case of similar, biotemplated ZnO surfaces, we concluded earlier that instead of hole capturing by water molecules to generate hydroxyl radicals (that in turn oxidize RhB) or electron capturing by the dissolved O_2_ from the conduction band to form reactive superoxide radical anions, the contribution of dye sensitization by RhB to photocatalytic degradation [37] was the likely scenario [38]. In addition to this earlier conclusion, the present series of experiments implies that the major route of RhB photodecomposition should be the reaction between the photoexcited RhB molecule and the n-type ZnO, since the p-type—and thus, electron injecting—Cu_2_O diminishes the photoreactivity against RhB.

The poor photocatalytic performance of pristine Cu_2_O nanoparticles can be attributed to the relatively short lifetime of the photoexcited carriers, as well as the hindered carrier diffusion towards the interface (as a consequence of, e.g., crystal defects or Cu vacancies) [59]. Thus, the effect of the direct contact with an n-type semiconductor (e.g., TiO_2_) has been widely studied on the extension of the carrier lifetime and hence the photocatalytic activity can be significantly improved [60,61]. Moreover, the increasing photocatalytic activity upon covering the Cu_2_O octahedra with ZnO can be explained based on the facet-dependent binding energy of the heterojunction: the (111) facet, which is dominant in an octahedral morphology, was found to be the highest in binding energy upon forming a heterojunction with an n-type semiconductor such as TiO_2_ [60]. The sufficiently formed Cu_2_O/ZnO heterojunction, thus, prolongs the charge carrier lifetime and allows the separation of photoexcited carriers.

Furthermore, despite the absorption of the Cu_2_O in the visible range, in contrast to the ZnO, it does not improve, but it decreases the photodecomposition of the dye by the butterfly wing. On the other hand, the right combination of wing–(15 nm ZnO)–Cu_2_O or NPs–(5 nm ZnO) is the best performing sample. This good performance is attributed both to the right spectral position of the reflectance maximum and to the efficient charge carrier separation taking place at the Cu_2_O–ZnO p-n junction [29,30,31,32,33,34,35,36]. The improvement in the reaction rate from sample #4 to sample #6 is attributed to the improvement in the electrical contact between Cu_2_O NPs and the ZnO following the deposition of the 5 nm ZnO adlayer.

The relative reaction rates of Figure 10 may be helpful in comparing the individual effects of the various components of the more complex biotemplated photonic nanoarchitectures that proved to have the best efficiency in the photodegradation of the dye. The reaction rate on bare glass is taken as unity. The ethanol pretreatment itself does not affect the photocatalytic behavior of the butterfly wing. The following sequence of the primary components: bare glass, Cu_2_O NPs, 15 nm ZnO layer on glass, and butterfly wing can be established: Cu_2_O NPs double the reaction rate compared to bare glass; the ZnO layer on glass increases the reaction rate to 2.5 times with respect to the glass, but the butterfly wings (both untreated and ethanol pretreated) exhibited a reaction rate approximately three times larger compared with glass. When comparing the biotemplated systems: (type-1 sample + Cu_2_O NPs) and the type-2 sample, the latter has a reaction rate twice that of the former. The (type-1 sample + Cu_2_O NPs) has a lower reaction rate than the butterfly wings. This decrease is attributed, on one hand, to the reduced reflectance by the spectral modifications induced by the Cu_2_O NPs (Figure 9, sample #3) and to the moderate catalytic compatibility between Cu_2_O and rhodamine B. The application of Cu_2_O on the type-2 sample does not produce an improvement in the reaction rate, likely because of the weak electric contact between the Cu_2_O and the ZnO. After this is corrected by the deposition of 5 nm ZnO layer, the best performing sample is obtained, with a reaction rate almost 4.3 times that of bare glass.

In Figure S4, the digital photographs and the measured reflectance of the samples after the first series of photocatalytic experiments are shown. The color shift of all the samples—visible to the naked eye—if compared with Figure 10 clearly shows that the dye is well adsorbed on the treated butterfly wings and persists there even after the gentle washing of the samples. The reflectance spectra quantitatively support this observation. The spectra show that indeed, as already reported in [37], the absorption of the dye in solution can significantly reduce the efficiency of the photocatalytic process. For this reason, the combination of the red edge of the photonic nanoarchitecture—to take advantage of the slow light effect—with the blue edge of the dye absorption can be the most advantageous spectral arrangement.

The above results demonstrate that properly chosen photonic nanoarchitectures of biologic origin—from the large “library” of such structures—in combination with well-chosen photocatalysts(s) can significantly enhance the efficiency of complex, hybrid biotemplated photonic/photocatalytic surfaces. Taken together, our findings suggest that the reason for the enhanced efficiency is complex; both the fast carrier separation at Cu_2_O-ZnO p-n heterojunctions and the slow light effect of the photonic nanoarchitecture are contributors. As discussed earlier, an advantageous overlap between the red edge of the reflectance maximum of the wing surface and the absorption of the test dye (RhB) is a prerequisite for an improved photocatalytic performance [37], and this can be achieved by a conformal ZnO coating in combination with Cu_2_O nanoparticles. On the contrary, when only Cu_2_O—an absorber in the visible light spectrum—was applied on a type-2 sample, no improvement in photocatalytic performance was observed. Only after this kind of sample was additionally coated by 5 nm of conformal ZnO was the improved photocatalytic performance observed. This supports that the main source of an improved photocatalytic performance by Cu_2_O NPs vs. a ZnO coating is a fast charge carrier separation at Cu_2_O–ZnO p-n junctions.

## 5. Conclusions

The possibilities of engineering the spectral and photocatalytic properties of biotemplated photonic nanoarchitectures based on the dorsal wing surfaces of male *Polyommatus icarus* butterflies were demonstrated experimentally. The spectral characteristics and the catalytic performance of complex photonic nanoarchitectures composed of three elements: blue wings of male *P. icarus* butterflies, Cu_2_O nanoparticles, and conformal ZnO nanolayers deposited by ALD, were studied. By applying different amounts of Cu_2_O NP sol in ethanol, monotonic changes were obtained in the spectral properties: the wavelength corresponding to the peak position of the reflectance maximum increased, while the intensity of the reflectance decreased. When the same treatment was applied on wings with a conformal coating of 15 nm ZnO, the modifications of the spectral parameters were much reduced. Furthermore, the distribution of the Cu_2_O NPs was uniform on the ZnO coated wings, while on the only ethanol pretreated wings, pronounced clustering was observed. Cu_2_O NPs enhance the photodecomposition of rhodamine B on the biotemplated photonic nanoarchitectures only in combination with ZnO and when good electrical contact is achieved between the Cu_2_O NPs and the ZnO. The biotemplated and spectrally engineered photonic nanoarchitecture based on the wings with conformally deposited 15 nm of ZnO had a reaction rate close to twice that obtained on the flat ZnO on glass. In this case, an approximately 4.3-fold enhancement compared with bare glass can be obtained in the photodecomposition reaction rate. The enhanced efficiency of the best performing hybrid nanoarchitectures is associated with slow light effects at the red edge of the reflectance maximum of the photonic nanoarchitectures and with fast carrier separation between the n-type ZnO and the p-type Cu_2_O. Well-chosen biotemplates, in combination with photocatalysts carefully selected to match the pollutant(s), may contribute to enhancing the efficiency of the photocatalytic process.

## Figures and Tables

**Table 1:** Reaction rates and relative reaction rates (with bare glass as unity) of the samples tested. Except for the comparison samples with simple structure (glass and glass + 15 nm ZnO, for these values are taken from [38]), averages for three independent photodecomposition experiments are shown. Note that a large volume of RhB solution (20 cm^3^) was used with a circular photocatalytic surface of 8 mm in diameter.

Sample Name	Reaction Rate [nmol/min]	Standard Deviation [nmol/min]	Relative Reaction Rate	Standard Deviation [%]
Glass	0.039	-	1	-
Glass + Cu_2_O	0.08	0.009	2.05	10.8
Glass + 15 nm ZnO	0.102	-	2.61	-
Type-1 (no pretreatment) (**#1**)	0.116	0.017	2.97	14.2
Type-1 (ethanol pretreatment) (**#2**)	0.119	0.017	3.05	14.6
Type-1 (eth.) + Cu_2_O (**#3**)	0.078	0.005	2	6.7
Type-1 (eth.) + Cu_2_O + 5 nm ZnO (**#5**)	0.091	0.02	2.33	22
Type-2	0.154	0.008	3.94	4.9
Type-2 + Cu_2_O (**#4**)	0.149	0.012	3.82	8.1
Type-2 + Cu_2_O + 5 nm ZnO (**#6**)	0.167	0.019	4.28	16.9

## Data Availability

All relevant data analyzed during this study are included in this published article and its Supplementary Information files. Raw datasets used during the current study are available from the corresponding author upon reasonable request.

## Supplementary Materials

The following supporting information can be downloaded at: https://www.mdpi.com/article/10.3390/nano12244490/s1, Figure S1: Uniformity of the spectral modifications produced by the deposition of 15 nm of conformal ZnO coating on the wings of male *Polyommatus icarus* butterflies in HSB visualization. The reddish wings are in pristine state, while all the others have received the coating in the same deposition run. The digital photographs have been processed as detailed in [49]. Figure S2: Optical micrographs in transmitted light and light transmittance through the samples prepared by drop drying Cu_2_O sol into the circular hole of the PTFE frames glued by PMMA onto glass and glass covered by 15 nm ZnO respectively. (a) 40 µL Cu_2_O sol on glass; (b) 40 µL Cu_2_O sol on ZnO covered glass; (c) transmittance measured with the integrating sphere on the sample in (a); (d) transmittance measured with the integrating sphere on the sample in (b), note in (d) the cutoff at 380 nm due to the ZnO layer, an additional spectrum is shown after increasing the amount of Cu_2_O on the sample; (e) the ratio of transmittances (glass + 40 µL Cu_2_O)/glass from (c), bellow 300 nm due to the low light intensity the noisy results are not shown; (f) transmittance measured with the illuminating and light collecting fibers in a collinear arrangement, with the sample in-between on the sample in (b), note in (f) the cutoff at 380 nm due to the ZnO layer, an additional spectrum is shown after increasing the amount of Cu_2_O on the sample. Figure S3: (a) Reflectance of filter paper loaded with Cu_2_O by placing the piece of paper in Cu_2_O sol stirred magnetically. Both sides of the loaded filter paper were measured, pristine filter paper was used as comparison. In (b), the decrease in reflectance due to the Cu_2_O loading is shown using the pristine paper as a reference; (c) the gradual decrease of reflectance of the same type of filter paper (kept in horizontal position) after loading on the upper surface 40, 80, and 120 µL of Cu_2_O sol using the pristine paper as a reference. Figure S4: Photographs (left) of the wing samples used (after the first run of measurements) in the photocatalytic experiments and the corresponding reflectance spectra (right): (1) type-1 sample, without ethanol pretreatment; (2) type-1 sample with ethanol pretreatment; (3) type-1 sample with 120 µL of Cu_2_O sol drop dried; (4) type-2 sample with 120 µL of Cu_2_O sol drop dried; (5) type-1 sample with 120 µL of Cu_2_O sol drop dried followed by the deposition of 5 nm of ZnO; (6) type-2 sample with 120 µL of Cu_2_O sol drop dried followed by the deposition of 5 nm of ZnO. The grey-shaded area marks the rhodamine B absorption.

## References

[1] Cheng P., Wang D., Schaaf P. (2022). A Review on Photothermal Conversion of Solar Energy with Nanomaterials and Nanostructures: From Fundamentals to Applications. Adv. Sustain. Syst..

[2] Ghosh S., Yadav R. (2021). Future of photovoltaic technologies: A comprehensive review. Sustain. Energy Technol. Assess..

[3] Rathore N., Panwar N.L., Yettou F., Gama A. (2021). A comprehensive review of different types of solar photovoltaic cells and their applications. Int. J. Ambient. Energy.

[4] Lianos P. (2017). Review of recent trends in photoelectrocatalytic conversion of solar energy to electricity and hydrogen. Appl. Catal. B Environ..

[5] Cushing S.K., Wu N. (2016). Progress and Perspectives of Plasmon-Enhanced Solar Energy Conversion. J. Phys. Chem. Lett..

[6] Zheng X., Zhang L. (2016). Photonic nanostructures for solar energy conversion. Energy Environ. Sci..

[7] Oelhafen P., Schüler A. (2005). Nanostructured materials for solar energy conversion. Sol. Energy.

[8] Ye W., Long R., Huang H., Xiong Y. (2017). Plasmonic nanostructures in solar energy conversion. J. Mater. Chem. C.

[9] Raja-Mogan T., Ohtani B., Kowalska E. (2020). Photonic Crystals for Plasmonic Photocatalysis. Catalysts.

[10] Liu J., Wu M., Van der Schueren B., Deparis O., Ye J., Ozin G.A., Hasan T., Su B.L. (2017). Slow Photons for Photocatalysis and Photovoltaics. Adv. Mater..

[11] Thomas A., Lethuillier-Karl L., Nagarajan K., Vergauwe R.M.A., George J., Chervy T., Shalabney A., Devaux E., Genet C., Moran J. (2019). Tilting a ground-state reactivity landscape by vibrational strong coupling. Science.

[12] Ebbesen T.W. (2016). Hybrid Light–Matter States in a Molecular and Material Science Perspective. Acc. Chem. Res..

[13] Kertész K., Piszter G., Horváth Z.E., Zámbó D., Deák A., Biró L.P. (2022). Effect of Plasmonic Au and Ag/Au Nanoparticles and Sodium Citrate on the Optical Properties of Chitin-Based Photonic Nanoarchitectures in Butterfly Wing Scales. Photonics.

[14] Cai Z., Li Z., Ravaine S., He M., Song Y., Yin Y., Zheng H., Teng J., Zhang A. (2021). From colloidal particles to photonic crystals: Advances in self-assembly and their emerging applications. Chem. Soc. Rev..

[15] Biró L.P., Vigneron J.P. (2011). Photonic nanoarchitectures in butterflies and beetles: Valuable sources for bioinspiration. Laser Photonics Rev..

[16] Bálint Z., Kertész K., Piszter G., Vértesy Z., Biró L.P. (2012). The well-tuned blues: The role of structural colours as optical signals in the species recognition of a local butterfly fauna (Lepidoptera: Lycaenidae: Polyommatinae). J. R. Soc. Interface.

[17] Stavenga D.G. (2014). Thin Film and Multilayer Optics Cause Structural Colors of Many Insects and Birds. Mater. Today Proc..

[18] Ingram A.L., Parker A.R. (2008). A review of the diversity and evolution of photonic structures in butterflies, incorporating the work of John Huxley (The Natural History Museum, London from 1961 to 1990). Philos. Trans. R. Soc. Lond. Ser. B Biol. Sci..

[19] Giraldo M.A., Stavenga D.G. (2016). Brilliant iridescence of Morpho butterfly wing scales is due to both a thin film lower lamina and a multilayered upper lamina. J. Comp. Physiol. A.

[20] Kinoshita S., Yoshioka S. (2005). Structural colors in nature: The role of regularity and irregularity in the structure. Chemphyschem.

[21] Wilts B.D., Giraldo M.A., Stavenga D.G. (2016). Unique wing scale photonics of male Rajah Brooke’s birdwing butterflies. Front. Zool..

[22] Mouchet S.R., Vukusic P. (2018). Structural Colours in Lepidopteran Scales. Adv. Insect Physiol..

[23] Czaplicki Z., Gliścińska E., Machnowski W. (2021). Natural Silk—An Unusual Fibre: Origin, Processing and World Production. Fibres Text. East. Eur..

[24] https://www.fibre2fashion.com/industry-article/6015/the-global-silk-industry.

[25] Piszter G., Kertész K., Sramkó G., Bálint Z., Biró L.P., Shaw J.A., Creath K., Lakshminarayanan V. (2022). Structural colors of blue butterflies: From photonic nanoarchitectures to DNA. Light in Nature IX.

[26] Huang M.H. (2019). Facet-Dependent Optical Properties of Semiconductor Nanocrystals. Small.

[27] Hezam A., Drmosh Q.A., Ponnamma D., Bajiri M.A., Qamar M., Namratha K., Zare M., Nayan M.B., Onaizi S.A., Byrappa K. (2022). Strategies to Enhance ZnO Photocatalyst’s Performance for Water Treatment: A Comprehensive Review. Chem. Rec..

[28] Kayani Z.N., Saleemi F., Batool I. (2015). Effect of calcination temperature on the properties of ZnO nanoparticles. Appl. Phys. A.

[29] He Z., Xia Y., Tang B., Jiang X., Su J. (2016). Fabrication and photocatalytic property of ZnO/Cu_2_O core-shell nanocomposites. Mater. Lett..

[30] Zhong S., Xiong D., Zhang B., Yang X., Yang T., Tian G., Zhang H., Yang W., Deng W. (2022). Structurally Unraveling the Photocarrier Behavior of Cu_2_O/ZnO Heterojunction Photodetectors. ACS Photonics.

[31] Norouzi A., Nezamzadeh-Ejhieh A., Fazaeli R. (2021). A Copper(I) oxide-zinc oxide nano-catalyst hybrid: Brief characterization and study of the kinetic of its photodegradation and photomineralization activities toward methylene blue. Mater. Sci. Semicond. Process..

[32] Kang Z., Yan X., Wang Y., Bai Z., Liu Y., Zhang Z., Lin P., Zhang X., Yuan H., Zhang X. (2015). Electronic Structure Engineering of Cu_2_O Film/ZnO Nanorods Array All-Oxide p-n Heterostructure for Enhanced Photoelectrochemical Property and Self-powered Biosensing Application. Sci. Rep..

[33] Lahmar H., Setifi F., Azizi A., Schmerber G., Dinia A. (2017). On the electrochemical synthesis and characterization of p-Cu_2_O/n-ZnO heterojunction. J. Alloy. Compd..

[34] Hussain S., Cao C., Nabi G., Khan W.S., Usman Z., Mahmood T. (2011). Effect of electrodeposition and annealing of ZnO on optical and photovoltaic properties of the p-Cu_2_O/n-ZnO solar cells. Electrochim. Acta.

[35] Guerguerian G., Elhordoy F., Pereyra C.J., Marotti R.E., Martín F., Leinen D., Ramos-Barrado J.R., Dalchiele E.A. (2012). ZnO/Cu_2_O heterostructure nanopillar arrays: Synthesis, structural and optical properties. J. Phys. D Appl. Phys..

[36] Cui J., Gibson U.J. (2010). A Simple Two-Step Electrodeposition of Cu_2_O/ZnO Nanopillar Solar Cells. J. Phys. Chem. C.

[37] Lim S.Y., Hedrich C., Jiang L., Law C.S., Chirumamilla M., Abell A.D., Blick R.H., Zierold R., Santos A. (2021). Harnessing Slow Light in Optoelectronically Engineered Nanoporous Photonic Crystals for Visible Light-Enhanced Photocatalysis. ACS Catal..

[38] Piszter G., Kertész K., Nagy G., Baji Z., Horváth Z.E., Bálint Z., Pap J.S., Biró L.P. (2022). Spectral tuning of biotemplated ZnO photonic nanoarchitectures for photocatalytic applications. R. Soc. Open Sci..

[39] Boriskina S.V. (2010). Photonic Molecules and Spectral Engineering. Springer Series in Optical Sciences.

[40] Reddy K.L., Mathew J.P., Shiby E., Kumar J. (2021). Spectral Engineering and Morphological Tuning of Amino Acid Capped Hydrophilic Upconversion Nanophosphors. J. Phys. Chem. C.

[41] Tóth E., Sipos Á., Fekete O.A., Csete M. (2021). Spectral Engineering Via Complex Patterns of Circular Nano-Object Miniarrays: II. Concave Patterns Tunable by Integrated Lithography Realized by Circularly Polarized Light. Plasmonics.

[42] Wiemers M., Stradomsky B.V., Vodolazhsky D.I. (2010). A molecular phylogeny of Polyommatus s. str. and Plebicula based on mitochondrial COI and nuclear ITS2 sequences (Lepidoptera: Lycaenidae). Eur. J. Entomol..

[43] Artemyeva E.A. (2005). Clinal Variation in Populations of the Common Blue Butterfly *Polyommatus icarus* Rott. (Lepidoptera, Lycaenidae). Russ. J. Genet..

[44] Barrington D., Pfeiffer B. (2021). *Polyommatus icarus* (European Common Blue) expnads into the United States. News Lepid. Soc..

[45] Rivest S.A., Kharouba H.M. (2021). Anthropogenic disturbance promotes the abundance of a newly introduced butterfly, the European common blue (*Polyommatus icarus*; Lepidoptera: Lycaenidae), in Canada. Can. J. Zool..

[46] Kertész K., Piszter G., Bálint Z., Biró L.P. (2019). Biogeographical patterns in the structural blue of male *Polyommatus icarus* butterflies. Sci. Rep..

[47] Piszter G., Kertész K., Bálint Z., Biró L.P. (2016). Variability of the Structural Coloration in Two Butterfly Species with Different Prezygotic Mating Strategies. PLoS ONE.

[48] Huang J.-Y., Madasu M., Huang M.H. (2018). Modified Semiconductor Band Diagrams Constructed from Optical Characterization of Size-Tunable Cu_2_O Cubes, Octahedra, and Rhombic Dodecahedra. J. Phys. Chem. C.

[49] Kertész K., Bálint Z., Piszter G., Horváth Z.E., Biró L.P. (2021). Multi-instrumental techniques for evaluating butterfly structural colors: A case study on *Polyommatus bellargus* (Rottemburg, 1775) (Lepidoptera: Lycaenidae: Polyommatinae). Arthropod Struct. Dev..

[50] Chang S., Yang X., Sang Y., Liu H. (2016). Highly Efficient Photocatalysts and Continuous-Flow Photocatalytic Reactors for Degradation of Organic Pollutants in Wastewater. Chem.-Asian J..

[51] McCullagh C., Skillen N., Adams M., Robertson P.K.J. (2011). Photocatalytic reactors for environmental remediation: A review. J. Chem. Technol. Biotechnol..

[52] Ghiradella H. (1994). Structure of butterfly scales: Patterning in an insect cuticle. Microsc. Res. Tech..

[53] Fang Y., Sun G., Cong Q., Chen G., Ren L. (2008). Effects of Methanol on Wettability of the Non-Smooth Surface on Butterfly Wing. J. Bionic Eng..

[54] Bixler G.D., Bhushan B. (2012). Bioinspired rice leaf and butterfly wing surface structures combining shark skin and lotus effects. Soft Matter.

[55] Fang Y., Sun G., Bi Y., Zhi H. (2015). Multiple-dimensional micro/nano structural models for hydrophobicity of butterfly wing surfaces and coupling mechanism. Sci. Bull..

[56] Kertész K., Baji Z., Deák A., Piszter G., Rázga Z., Bálint Z., Biró L.P. (2021). Additive and subtractive modification of butterfly wing structural colors. Colloid Interface Sci. Commun..

[57] Deparis O., Mouchet S.R., Su B.-L. (2015). Light harvesting in photonic crystals revisited: Why do slow photons at the blue edge enhance absorption?. Phys. Chem. Chem. Phys..

[58] Zhao H., Hu Z., Liu J., Li Y., Wu M., Van Tendeloo G., Su B.-L. (2018). Blue-edge slow photons promoting visible-light hydrogen production on gradient ternary 3DOM TiO_2_-Au-CdS photonic crystals. Nano Energy.

[59] Borgwardt M., Omelchenko S.T., Favaro M., Plate P., Höhn C., Abou-Ras D., Schwarzburg K., van de Krol R., Atwater H.A., Lewis N.S. (2019). Femtosecond time-resolved two-photon photoemission studies of ultrafast carrier relaxation in Cu_2_O photoelectrodes. Nat. Commun..

[60] Nie J., Yu X., Hu D., Wang T., Liu Z., Zhao N., Li J., Yao B. (2020). Preparation and Properties of Cu_2_O/TiO_2_ Heterojunction Nanocomposite for Rhodamine B Degradation under Visible Light. ChemistrySelect.

[61] Koiki B.A., Arotiba O.A. (2020). Cu_2_O as an emerging semiconductor in photocatalytic and photoelectrocatalytic treatment of water contaminated with organic substances: A review. RSC Adv..

