# Supplementary material for: Spectral Engineering of Hybrid Biotemplated Photonic/Photocatalytic Nanoarchitectures
Source: Nanomaterials (Basel). 2022 Dec 19;12(24):4490. doi: 10.3390/nano12244490 (PMC9782751; doi:10.3390/nano12244490)
Supplement: Supplementary file 1 [file nanomaterials-12-04490-s001.zip › nanomaterials-2097299-supplementary.pdf]

Supplementary Information

on

Spectral Engineering of Hybrid Biotemplated Photonic/Photocatalytic Nanoarchitectures

by

Gábor Piszter, Krisztián Kertész, Dávid Kovács, Dániel Zámbó, Zsófia Baji, Levente Illés, Gergely Nagy, József Sándor Pap, Zsolt Bálint, László Péter Biró

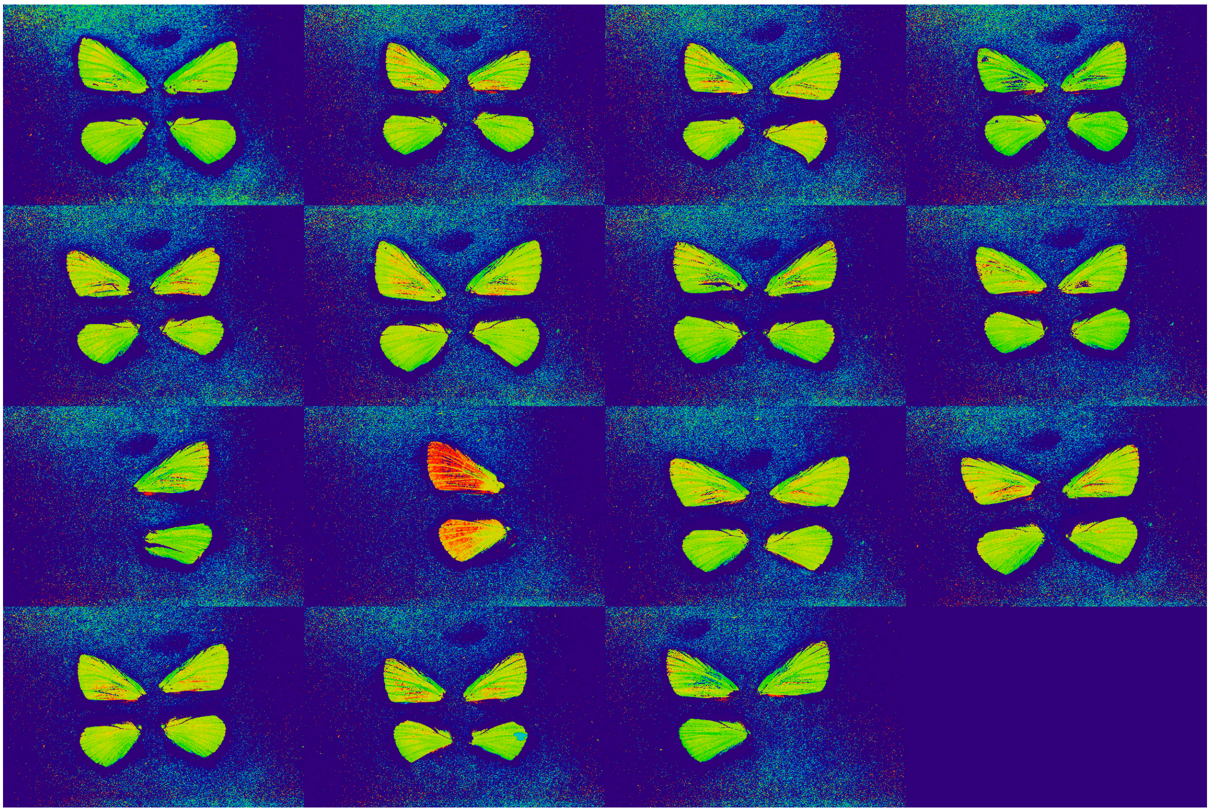

Figure S1. Uniformity of the spectral modifications produced by the deposition of 15 nm of conformal ZnO coating on the wings of male *Polyommatus icarus* butterflies in HSB visualization. The reddish wings are in pristine state, while all the others have received the coating in the same deposition run. The digital photographs have been processed as detailed in [49].

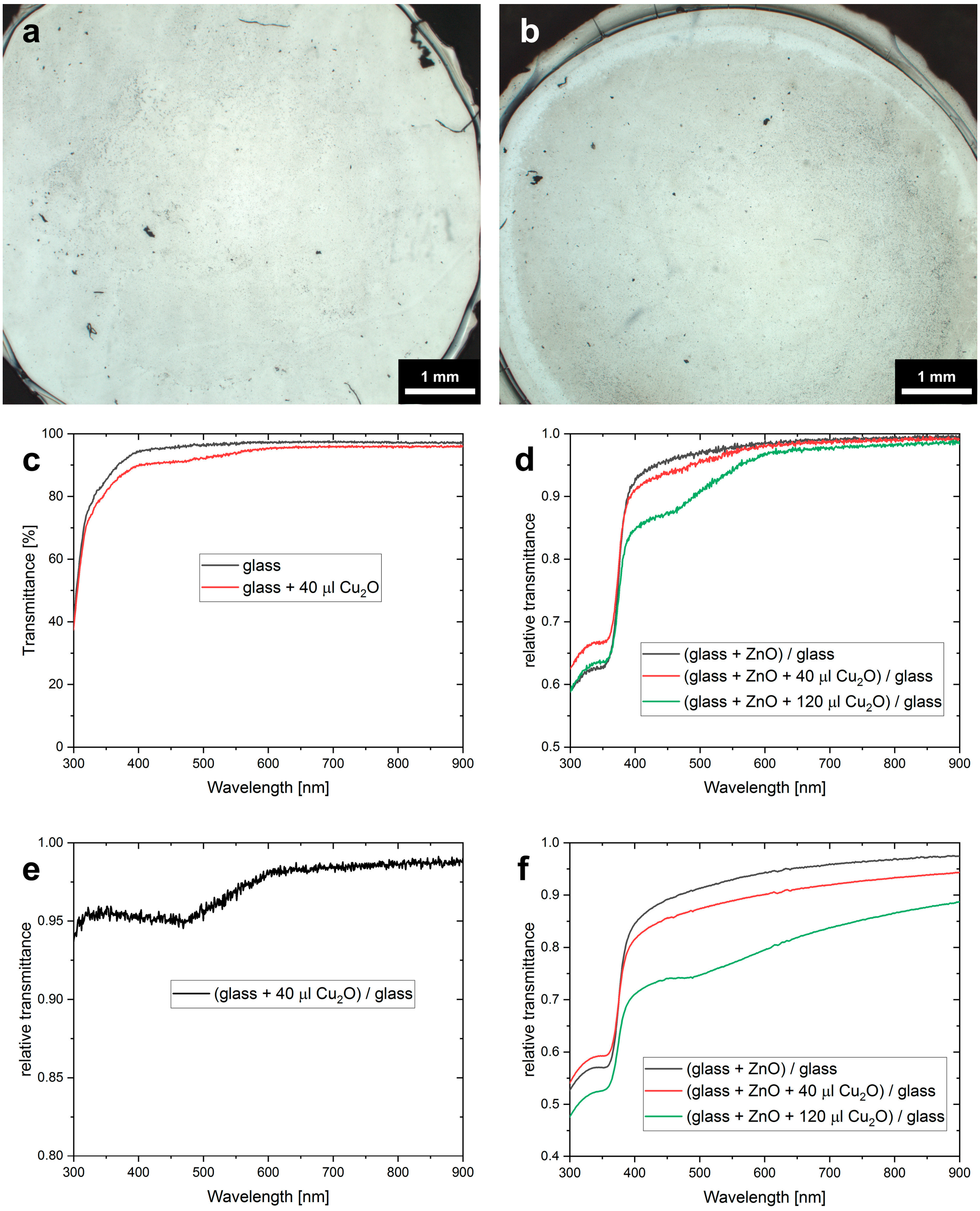

Figure S2. Optical micrographs in transmitted light and light transmission through the samples prepared by drop drying Cu_2O sol into the circular hole of the PTFE frames glued by PMMA onto glass and glass covered by 15 nm ZnO respectively. (**a**) 40 µl Cu_2O sol on glass; (**b**) 40 µl Cu_2O sol on ZnO covered glass; (**c**) transmittance measured with the integrating sphere on the sample in (**a**); (**d**) transmittance measured with the integrating sphere on the sample in (**b**), note in (**d**) the cutoff at 380 nm due to the ZnO layer, an additional spectrum is shown after increasing the amount of Cu_2O on the sample; (**e**) the ratio of transmittances (glass + 40 µl Cu_2O)/glass from (**c**), bellow 300 nm due to the low light intensity the noisy results are not shown; (**f**) transmittance measured with the illuminating and light collecting fibers in a collinear arrangement, with the sample in-between on the sample in (**b**), note in (**f**) the cutoff at 380 nm due to the ZnO layer, an additional spectrum is shown after increasing the amount of Cu_2O on the sample.

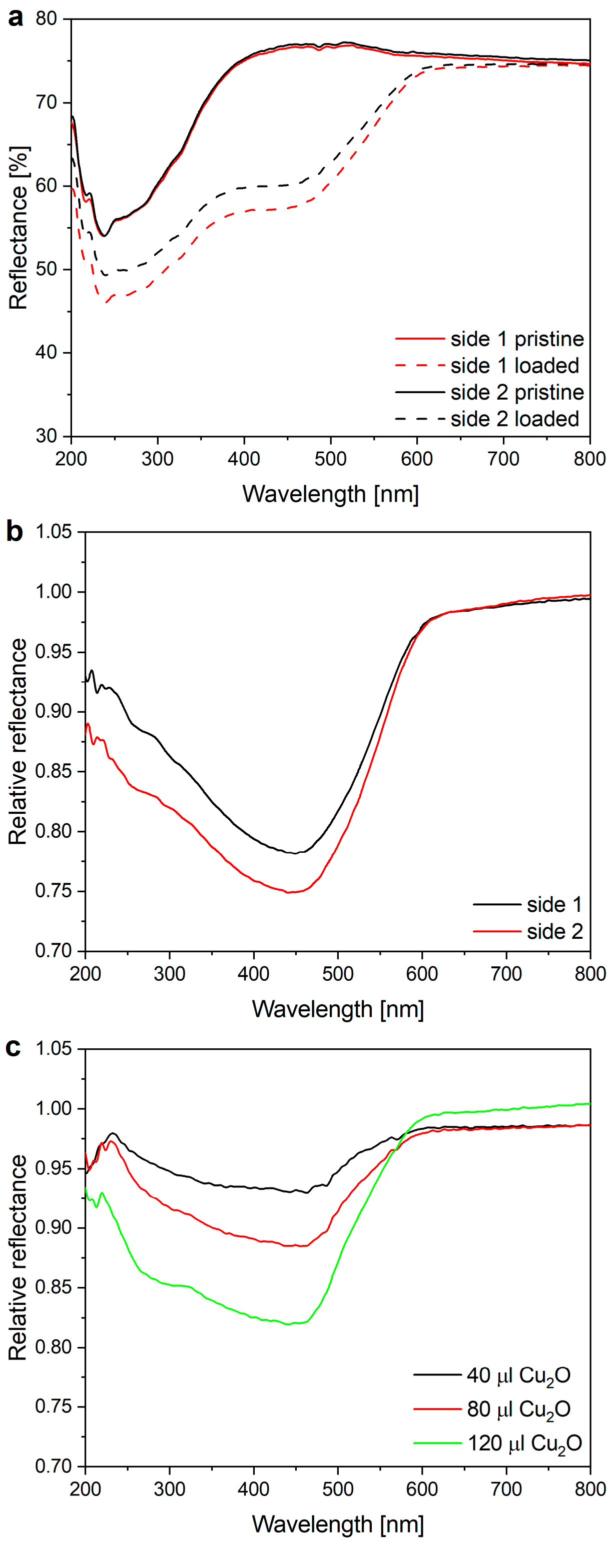

Figure S3. (**a**) Reflectance of filter paper loaded with Cu_2O by placing the piece of paper in Cu_2O sol stirred magnetically. Both sides of the loaded filter paper were measured, pristine filter paper was used as comparison. In (**b**), the decrease in reflectance due to the Cu_2O loading is shown using the pristine paper as a reference; (**c**) the gradual decrease of reflectance of the same type of filter paper (kept in horizontal position) after loading on the upper surface 40, 80, and 120 µl of Cu_2O sol using the pristine paper as a reference.

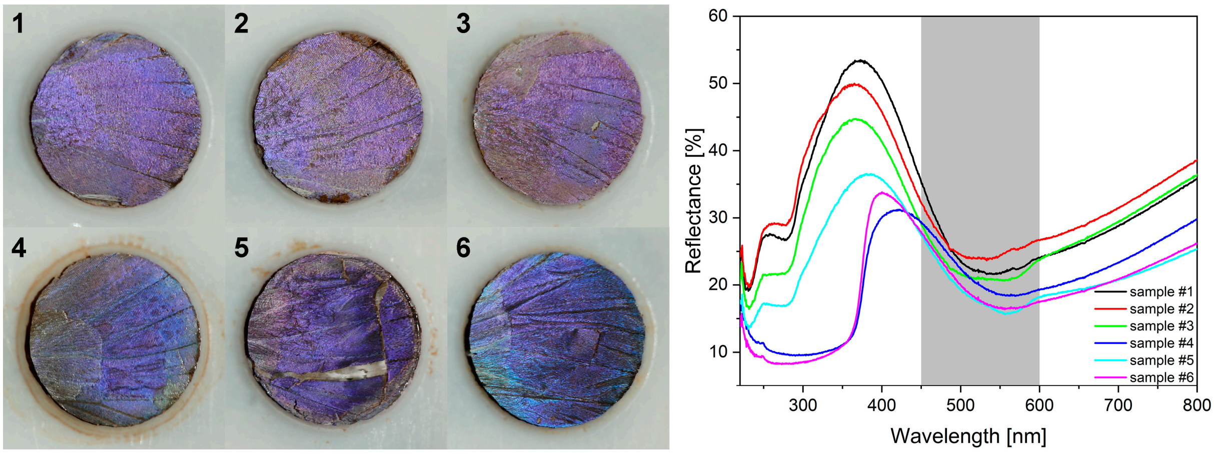

Figure S4. Photographs (left) of the wing samples used (after the first run of measurements) in the photocatalytic experiments and the corresponding reflectance spectra (right): (**1**) type-1 sample, without ethanol pretreatment; (**2**) type-1 sample with ethanol pretreatment; (**3**) type-1 sample with 120 µl of Cu_2O sol drop dried; (**4**) type-2 sample with 120 µl of Cu_2O sol drop dried; (**5**) type-1 sample with 120 µl of Cu_2O sol drop dried followed by the deposition of 5 nm of ZnO; (**6**) type-2 sample with 120 µl of Cu_2O sol drop dried followed by the deposition of 5 nm of ZnO. The grey-shaded area marks the rhodamine B absorption.
